# ATR-p53 Restricts Homologous Recombination in Response to Replicative Stress but Does Not Limit DNA Interstrand Crosslink Repair in Lung Cancer Cells

**DOI:** 10.1371/journal.pone.0023053

**Published:** 2011-08-12

**Authors:** Bianca M. Sirbu, Sarah J. Lachmayer, Verena Wülfing, Lara M. Marten, Katie E. Clarkson, Linda W. Lee, Liliana Gheorghiu, Lee Zou, Simon N. Powell, Jochen Dahm-Daphi, Henning Willers

**Affiliations:** 1 Laboratory of Cellular and Molecular Radiation Oncology, Massachusetts General Hospital Cancer Center, Charlestown, Massachusetts, United States of America; 2 Department of Radiation Oncology, University of Hamburg, Hamburg, Germany; 3 Center for Cancer Research, Massachusetts General Hospital Cancer Center, Charlestown, Massachusetts, United States of America; 4 Department of Radiation Oncology, Memorial Sloan Kettering Cancer Center, New York, New York, United States of America; 5 Institute of Radiobiology and Molecular Radiation Oncology, University of Marburg, Marburg, Germany; 6 Harvard Medical School, Boston, Massachusetts, United States of America; University of Medicine and Dentistry of New Jersey, United States of America

## Abstract

Homologous recombination (HR) is required for the restart of collapsed DNA replication forks and error-free repair of DNA double-strand breaks (DSB). However, unscheduled or hyperactive HR may lead to genomic instability and promote cancer development. The cellular factors that restrict HR processes in mammalian cells are only beginning to be elucidated. The tumor suppressor p53 has been implicated in the suppression of HR though it has remained unclear why p53, as the guardian of the genome, would impair an error-free repair process. Here, we show for the first time that p53 downregulates foci formation of the RAD51 recombinase in response to replicative stress in H1299 lung cancer cells in a manner that is independent of its role as a transcription factor. We find that this downregulation of HR is not only completely dependent on the binding site of p53 with replication protein A but also the ATR/ATM serine 15 phosphorylation site. Genetic analysis suggests that ATR but not ATM kinase modulates p53's function in HR. The suppression of HR by p53 can be bypassed under experimental conditions that cause DSB either directly or indirectly, in line with p53's role as a guardian of the genome. As a result, transactivation-inactive p53 does not compromise the resistance of H1299 cells to the interstrand crosslinking agent mitomycin C. Altogether, our data support a model in which p53 plays an anti-recombinogenic role in the ATR-dependent mammalian replication checkpoint but does not impair a cell's ability to use HR for the removal of DSB induced by cytotoxic agents.

## Introduction

Genetic exchanges mediated by homologous DNA sequences must be tightly regulated to maintain genomic stability [1]. An active homologous recombination (HR) pathway is needed for the repair and restart of collapsed DNA replication forks [2]. Cells with defects in HR are impaired in their ability to remove DNA interstrand crosslinks (ICL) as produced for example by mitomycin C (MMC). DNA double-strand breaks (DSB) occurring in S-phase post-replication or in G2 are repaired by HR in a typically error-free manner because homologous DNA sequence on the sister chromatid can serve as an accurate template for repair. In contrast, spontaneous DNA exchanges between homologous sequences in mitotically growing cells have to be limited and HR activities at stalled replication forks may not always be desirable [1], [3], [4]. The anti-recombinogenic factors that restrict HR in mammalian cells are only beginning to be elucidated.

The p53 tumor suppressor plays a pivotal role in the maintenance of genomic stability and suppression of cellular transformation [1], [5]. As a consequence, wild-type p53 function is disrupted by genetic mutations or other mechanisms in the majority, if not all, human cancers [5]. p53 has emerged as a multifunctional regulator, which is at the center of several pathways involved in apoptosis, cell-cycle control, and DNA repair. Many functions of p53 are mediated by transcriptional activation of downstream target genes [6]. We and others have established an additional transactivation-independent role of p53 in the suppression of HR processes across a variety of cell systems and assays [7], [8], [9], [10], [11], [12], [13]. For example, several p53 mutations such as L22Q/W23S (p53QS), A138V, or V143A, which impair p53's ability to transactivate target genes including p21, do not compromise p53's ability to downregulate HR [10], [14]. p53 appears to affect HR through various direct protein and DNA interactions [8], [15], [16], [17], [18], [19], [20], [21], [22], [23]. A direct interaction with the single-stranded (ss) DNA-binding Replication Protein A (RPA) appears to inhibit HR at an early step, and additional interactions with BRCA2 and RAD51 may serve the same purpose [9], [10], [24]. Downregulation of HR is dependent upon intact core and tetramerization domains of p53 [7], [8], [12], while the C-terminal end appears dispensable [12], [13]. The upstream factors that regulate p53-mediated HR suppression remain largely unknown [25].

Multiple observations link p53 directly to DNA replication. p53 co-localizes with sites of replication [26], [27], is expressed in parallel to DNA synthesis when cells reenter the cell-cycle [28], and migrates into the nucleus in S-phase [29], [30]. Replication of damaged DNA is blocked by p53 *in-vitro* [31]. Following inhibition of replication elongation by hydroxyurea (HU), transcriptionally inactive p53 accumulates in S-phase [32]. Consistent with these data, p53 downregulates HR if replication elongation is blocked [11], [33]. What has remained unknown is whether p53's wild-type transactivation activity is required for its suppressive role in replication-associated HR.

P53 is phosphorylated directly or indirectly by the ATM (Ataxia Telangiectasia Mutated) and ATR (ATM and Rad3-related) kinases [34], [35], but the functional consequences of these modifications with regard to HR regulation have not been established. ATM responds primarily to DSBs and phosphorylates a network of substrates [36]. ATM affects both HR as well as error-prone and error-free non-homologous end-joining [37], [38], [39]. The ATR kinase plays a central role in the response to replicative stress, and the phosphorylation of ATR substrates collectively inhibits replication and maintains replication forks, thereby preventing genomic instability [40], [41]. Importantly, HR is used to re-initiate replication but may also cause inappropriate strand-exchange events at stalled forks if not regulated properly [40], [42]. Compared to yeast, the anti-recombinogenic functions of the replication checkpoint in mammalian cells are poorly understood [40], [42].

Here, we demonstrate for the first time that transactivation-deficient p53 downregulates HR in response to replicative stress. We establish that HR suppression by p53 occurs within only hours of replicative stress and is dependent on both, the RPA binding site and ATR phosphorylation site serine 15, thus placing p53 into the mammalian replication checkpoint. In contrast to p53's role in the replicative stress response, the suppression of homology-mediated repair of directly or indirectly induced DSB appears relaxed, consistent with p53's role as a guardian of the genome.

## Results

### Differential regulation of HR by transactivation-impaired p53

**Figure 1:**
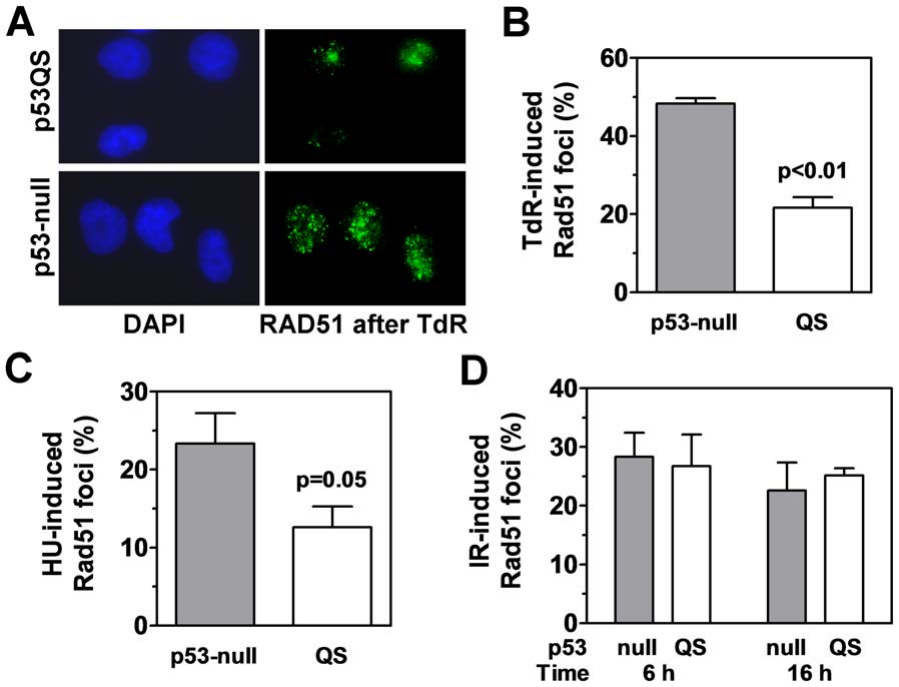
Transactivation-impaired p53 restricts subnuclear RAD51 foci formation in response to replication stress. (A) Representative images of subnuclear RAD51 foci formation in H1299 cells stably expressing p53QS or p53-null cells treated with 5 mM thymidine (TdR) for 24 hours. (B) Impact of p53 status (null versus QS) on RAD51 foci formation in H1299 cells treated with 5 mM TdR for 24 hours. Bars represent mean with standard error based on 3 independent repeats. (C) Impact of p53 status on RAD51 foci formation in H1299 cells treated with 1 mM hydroxyurea (HU) for 24 hours. Bars represent mean with standard error based on 5 independent repeats. (D) Impact of p53 status on RAD51 foci formation in H1299 cells 6 or 16 hours (h) after treatment with 2 Gy ionizing radiation (IR). Bars represent mean with standard error based on 2–5 independent repeats. All y-axes indicate percentage of treated cells with at least 10 RAD51 foci per nucleus after subtracting the percentage of untreated cells with background levels of RAD51 foci. P-values are based on Student's t-test (two-tailed).

It has been previously shown that p53 suppresses HR following induction of replicative stress [11], [33]. However, it was unknown whether p53's transactivation activity is required for this function. To address this question, we utilized p53-null cells stably transfected with a previously characterized transactivation-impaired p53 mutant, p53QS [10]. We induced the formation of subnuclear RAD51 foci by treatment of cells with inhibitors of replication elongation, thymidine and HU (Figure 1A, and data not shown). In response to either drug, there was a statistically significant suppression of RAD51 foci formation in p53QS-expressing cells, compared to p53-null controls (Figure 1BC, Figure S1A). As a control, the magnitude of this effect was similar to the HR suppressing ability of endogenous wild-type p53, although this experiment was performed in a different cell line, A549 (Figure S1B). In contrast, Figure 1D shows that p53QS did not modulate RAD51 foci induction in cells exposed to ionizing radiation (IR), which produces DSB throughout the cell cycle, with sister chromatid DSB occurring post-replication and in G2 repaired by HR.

**Figure 2:**
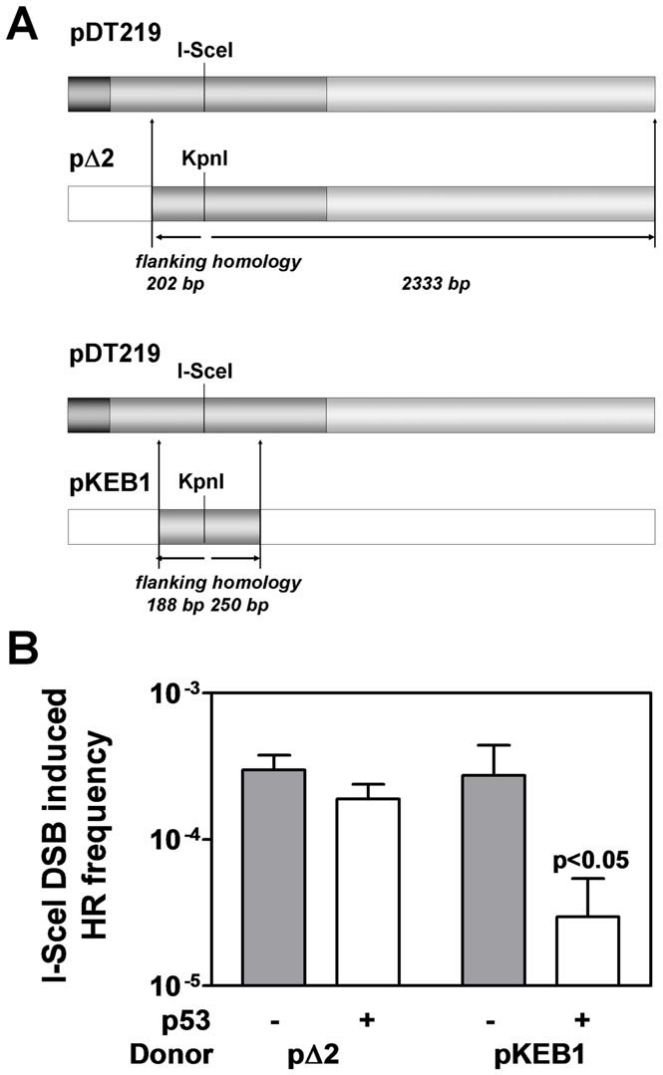
p53 has a differential effect on I-SceI-induced HR in the chromosomal pDT219 recombination substrate. (A) Plasmid substrate pDT219 carries a copy of the bacterial gpt gene inactivated by insertion of an I-SceI recognition site into the unique KpnI site of pSV2-gpt. HR events were induced in 10.1 mouse embryo fibroblasts carrying pDT219 by co-transfection of an I-SceI expression vector and a homologous donor. pΔ2 is characterized by 202 bp and 2333 bp of uninterrupted sequence homology shared with pDT217, while pKEB1 only contains 188 bp and 250 bp of homology flanking the break site. Following co-transfection of pKEB1 or pΔ2 together with an I-SceI expression vector, recombinants were scored in a colony formation assay. (B) I-SceI-induced HR frequencies obtained with chromosomally integrated pDT219 are plotted against p53 status, (−) indicating p53-null, (+) indicating the of transactivation-deficient p53-A135V mutant which is functionally analogous to p53QS. Bars represent the geometric mean with SEM of 3–6 independent experiments. The relative suppression of HR in the presence of p53-A135V compared to p53-null cells is indicated for each of the two donor plasmids. The relative suppression of HR was compared using the unpaired t-test (two-tailed).

To model DSB repair on substrates resembling aligned sister chromatids, we modified a previously used recombination assay that renders cells resistant to mycophenolic acid upon successful HR. The bacterial *gpt* gene in the recombination substrate pDT219 was inactivated by insertion of an I-SceI recognition site into the KpnI site (Figure 2A). Adapting a previously characterized murine model to study the transactivation-independent properties of p53 [13], we expressed transactivation-impaired p53-A135V in mouse embryonic fibroblasts (MEFs) carrying the pDT219 substrate which harbors a recognition site for the rare-cutting site-directed I-SceI meganuclease (data not shown). We previously showed that this p53 mutant is capable of suppressing spontaneous HR events, analogously to p53QS in human cells [10], [13]. We first assessed the effect of this mutant to suppress DSB-induced HR using the homologous donor sequence pΔ2, which is co-transfected an I-SceI meganuclease expression vector. In this system, homology-mediated repair is mediated by stretches of uninterrupted homology of 202 bp and 2,333 bp upstream and downstream of the I-SceI site, respectively. We did not detect a statistically significant difference in DSB-induced HR frequencies between cells with and without p53-A135V (Figure 2B). There was no difference in transfection efficiencies between the different clones (data not shown). Next, we modified the donor plasmid to reduce the length of shared sequence homology to only 188–250 bp (pKEB1). With this modification, the suppressive effect of p53 was statistically significantly increased to 10-fold (p<0.01). Similarly, in a commonly used GFP-based recombination substrate, pDR-GFP, in which HR is mediated by approximately 400 bp of shared uninterrupted sequence homology flanking the I-SceI site, transactivation-impaired human or murine p53 suppressed DSB-induced HR by several fold (Figure S2).

Together, these data suggest that transactivation-impaired p53 downregulates HR in response to replicative stress but does not affect homology-mediated repair of DSBs if the length of shared homology exceeds >250–400 bp as would be typical for exchanges between sister chromatids. The observed suppression of DSB-induced HR in the presence of short homologies may be unrelated to p53's role in regulating replication-associated HRR and was not pursued further.

### HR suppression requires the serine 15 site of p53

**Figure 3:**
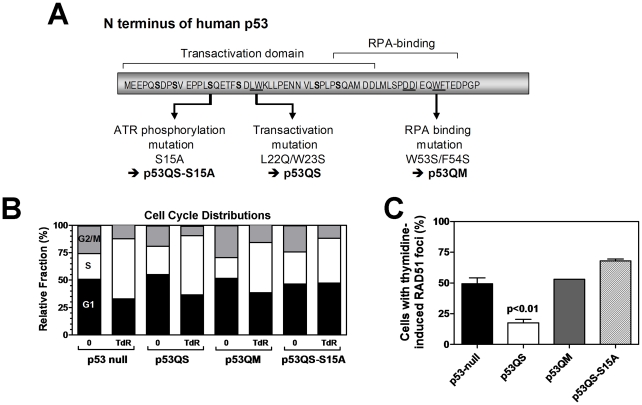
Genetic analysis of p53's effect on HR in response to replicative stress reveals a role of the ATM/ATR phosphorylation site serine 15 (S15). (A) Illustration of N-terminal p53 mutations introduced by site-directed mutagenesis. The mutant constructs were stable expressed from a common chromosomal integration site in H1299 cells (see Materials and Methods). (B) Cell cycle distributions for H1299 clones stably expressing a p53 N-terminal mutant. TdR, 5 mM thymidine for 24 hours. (C) Effect of p53 status on thymidine induced RAD51 foci formation, analogously to the experiments shown in Figure 1. p-value, result of t-test (two-tailed) comparing p53QS to p53-null cells.

In response to replication fork stalling, p53 is phosphorylated at serine 15 (Figure S3A,B) [34], [43]. However, the functional consequences of this modification were unknown. We created a phospho-mutant of p53QS by introducing a serine 15 to alanine mutation (p53QS-S15A) (Figure 3A). We also generated a RPA-binding mutant of p53 (p53QM) by additionally mutating amino acids 53 and 54, which were previously shown to be important for HR suppression [10]. When stably expressed in p53-null cells (Figure S4), all of these mutants were associated with similar cell cycle profiles in response to thymidine treatment (Figure 3B). As predicted, p53QM was unable to suppress RAD51 foci formation in thymidine-treated cells, and this was observed in multiple subclones (Figure 3C, and data not shown). Strikingly, blocking serine 15 phosphorylation also completely abrogated the ability of p53QS to downregulate RAD51 foci.

**Figure 4:**
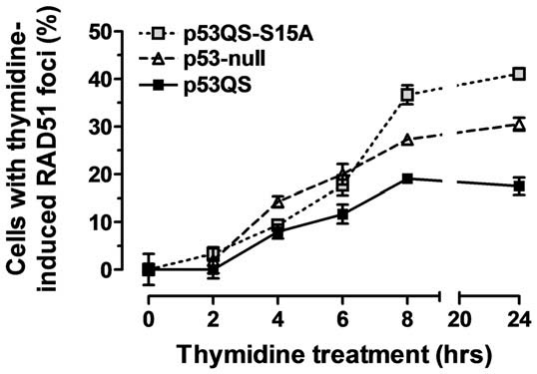
Kinetics of RAD51 foci formation reveals early suppressive effect of p53 in response to replication stalling. The time course of induced RAD51 foci in thymidine treated H1299 clones was measured analogously to the experiments shown in Figure 1.

To assess how early in the response to replicative stress the S15 site of p53 is required, we studied the kinetics of foci formation. Figure 4 shows that RAD51 foci formation was already impaired within 6 hours of thymidine treatment in p53-null and p53QS-S15A expressing cells. Consistent with this observation, S15 phosphorylation in p53QS expressing cells was observed within 6 hours of treatment (Figure S3C). Furthermore, p53QS suppressed the local accumulation of RPA at 6 hours, consistent with prior data on p53's ability to inhibit RPA binding to single-stranded DNA, which is a necessary step for the initiation of HR (Figure S5).

### Dependence of p53-mediated HR suppression on ATR

**Figure 5:**
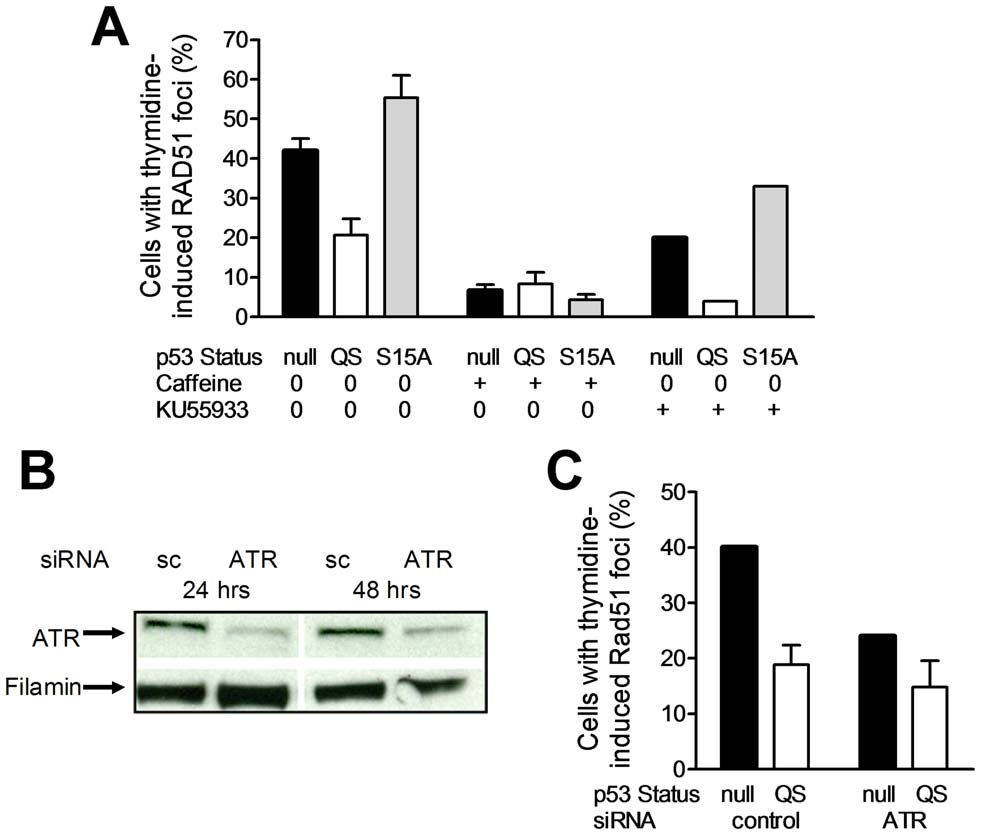
Implicating ATR in the p53-mediated suppression of HR. (A) H1299 clones were treated with thymidine (5 mM for 24 hours) with or without concurrent caffeine (5 µM) or KU55933 (20 µM) treatment. (B) Western blot illustrating siRNA mediated depletion of ATR in H1299 cells. sc, scrambled siRNA control. (C) Effect of p53QS status and ATR depletion on RAD51 foci induction, measured analogously to Figure 1.

The S15 site of p53 is modified by ATM and ATR kinases [34], [44], [45], and both kinases have been shown to promote HR in cells with impaired or lost wild-type p53 function [39], [46], [47]. As expected, upon treatment with caffeine, which inhibits ATR as well as ATM, the ability of cells to form RAD51 foci in response to thymidine was greatly diminished (Figure 5A). Strikingly, the ability of p53QS to reduce RAD51 formation compared to p53-null or p53QS-S15A cells was abrogated. To distinguish between the function of ATM versus ATR, we treated cells with the ATM inhibitor KU55933. In this setting, the HR suppressive effect of p53QS was preserved, indicating a dependence on ATR rather than ATM. To confirm this finding, we treated cells with siRNA directed against ATR as no specific ATR inhibitor is available. Sufficient ATR protein depletion was achieved following double siRNA transfection, and cells retained normal growth during the 48-hour duration of the experiment (Figure 5B, and data not shown). As observed previously, there was a p53-independent reduction of HR in ATR siRNA treated cells: the percentage of RAD51 foci positive p53-null cells was reduced by 16% compared to cells transfected with control siRNA, i.e., from 40% to 24% (Figure 4C). Compared to control siRNA transfected cells, the relative p53-mediated suppression of HR in ATR siRNA transfected cells was less pronounced though not completely abrogated which is consistent with residual p53QS function. Altogether, these data suggest that ATR regulates HR through p53-dependent and -independent mechanisms.

### p53 does not compromise the RAD51 response to DSB after thymidine or MMC

**Figure 6:**
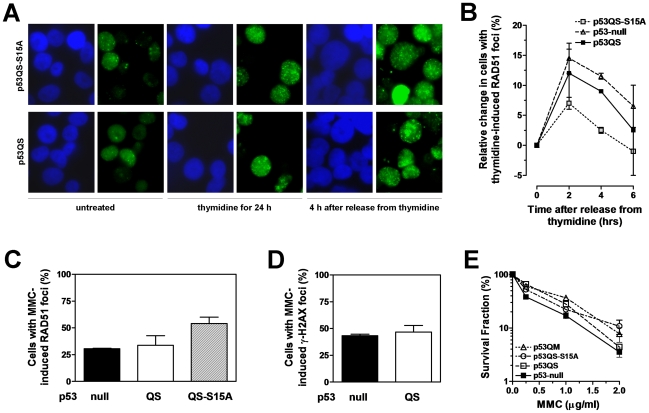
HR suppressive function of p53 is bypassed in the cellular response to DSB. (A) Staining for γ-H2AX as a marker of DSB formation, illustrating increase in DSB in both H1299 clones within 4 hours after release from thymidine (5 mM for 24 hours). (B) Time course of RAD51 foci induction, analogously to Figure 4, following removal of thymidine. To illustrate the similar increase in RAD51 foci induction irrespective of p53 status, the percentage of cells with foci was normalized to 0 at time 0 hours (h), i.e., at time of removal of thymidine. (C) Impact of p53 status on RAD51 foci induced 4 hours after treatment with mitomycin C (MMC) (0.5 µg/ml for 1 hour). Y-axis indicates percentage of cells with at least 10 induced RAD51 foci per nucleus. Similar results were seen after 24 hours (data not shown). (D) Impact of p53 status on γ-H2AX foci formation 24 hours after treatment with MMC. Y-axis indicates percentage of cells with at least 20 induced foci per nucleus. (E) Clonogenic survival of H1299 clones with varying p53 status. All data points are based on 2–3 independent repeat experiments.

HR is utilized for replication fork repair and restart [2], a process that should not be opposed by p53 as it is required for maintenance of genomic stability and cell survival. Upon release from a 24-hour incubation with thymidine (as shown in Figure 4), we observed an increase in γ-H2AX foci, consistent with the occurrence of DSB at collapsed replication forks (Figure 6A). There was a similar relative increase in RAD51 foci that was independent of p53 status and consistent with HR-mediated fork restart (Figure 6B). Therefore, in this setting, p53QS did not exert a suppressive effect on RAD51 foci formation.

We also exposed cells to the crosslinking agent MMC, which leads to the generation of DSB at collapsed replication forks. Consistent with the data in Figure 6B, p53QS did not suppress RAD51 foci formation in response to MMC (Figure 6C). Importantly, there was no difference in residual γ-H2AX foci in p53-null and p53QS expressing cells 24 hours after MMC exposure, suggesting that p53 does not compromise DSB repair (Figure 6D). Lastly, expression of p53QS did not impair the survival of MMC-treated cells consistent with the similar RAD51 and γ-H2AX foci levels -expressing cells (Figure 6E). To the contrary, there was a slight but robust increase in resistance to MMC upon expression of any of the p53 mutant forms. Interestingly, a similar outcome was seen when expressing the p53-A135V mutant in mouse embryonic fibroblasts (Figure S6). The mechanisms by which transactivation-inactive p53 may promote MMC survival remain to be determined (see Discussion).

## Discussion

While p53's role as a transcription factor that controls apoptosis and cell cycle progression is firmly established, a myriad of studies over the past >15 years has ascribed a multitude of additional biochemical and cellular functions to p53 [1], [6]. A transactivation-independent role of p53 in the downregulation of HR has been reproducibly described by several laboratories, including our own [7], [8], [10], [14], [48]. Because careful control of HR activities is important for the response to stalled or collapsed replication forks, elucidating the role of p53 in HR is critical for a better understanding of tumor initiation and progression.

We show here for the first time that p53 downregulates HR in response to replicative stress in a manner that is independent of its role as a transcription factor (Figures 1, 2, 3). Our data are consistent with the idea that p53's role in HR is dependent on interactions with RPA and ATR kinase, thus implicating p53 in the ATR replication checkpoint (Figure 3, 5). Overall, the anti-recombinogenic functions of the replication checkpoint remain to be fully established [40], [49]. In fission yeast, the Chk1 homologue inhibits Mus81 and Rad60 function, thereby preventing undesired recombination [50], [51]. In higher eukaryotes, ATR phosphorylates BLM, a known anti-recombinogenic factor [52], [53]. On the other hand, ATR has been shown to promote HR [46], [47]. Consistent with these data, our findings imply that both ATR and ATM promote RAD51 foci formation in response to replicative stress in a p53-independent fashion (Figure 5). Thus, there may exist a positive and negative (via p53) regulation of HR by ATR.

With regard to potential limitations of our work, an inherent limitation of foci studies is that they cannot directly measure protein activities at replication forks (Figure 1, 3, 4). However, foci endpoints are widely used in the literature to determine molecular mechanisms and genetic determinants of HR [15], [46], [54]. Second, a similar limitation applies to our plasmid system (Figure 2), which may not be an accurate measure of physiological HR events that are under p53 control. Third, while all of our and other data suggest that the human p53QS mutant and mouse p53-A135V (or human homolog) are functionally equivalent in terms of suppressing HR in a transactivation-independent manner (for example, Figure S2) [7], [10], [12], [13], we cannot exclude the possibility that unknown differences may exist. Lastly, we also caution that results obtained with one cell line, such as H1299 lung cancer cells in this study, may not be readily generalized to other cell lines.

What are the molecular mechanisms by which S15 phosphorylation of p53 could suppress HR? In a previously published model, sequestration of RPA from ssDNA will inhibit the subsequent loading of RAD51, and thus is one means by which p53 suppresses HR [10]. The p53 N-terminus competes with ssDNA for the OB-fold domain of RPA1's N-terminus [55]. Thus, we speculate that mechanisms may exist by which N-terminal phosphorylation of p53 promotes the binding to RPA1, thereby affecting the ssDNA-binding affinity of the DNA binding domains of RPA. For example, altered ssDNA-RPA binding could lead to unscheduled release of RPA from ssDNA impairing with proper RAD51 loading, or p53 may trap RPA on ssDNA and delay RAD51 loading. There are strong interdependencies between the N-terminal p53 phosphorylation sites [56], [57]. In H1299 cells, mutating S15 leads to reduced S37 phosphorylation after irradiation [57]. Interestingly, Lowry et al. recently found evidence of a collapsed region in the intrinsically unstructured p53 domain with a loop structure centered around residues 34–36 [58]. These authors suggested that S37 phosphorylation may lead to an open conformation of this domain and thereby promote binding to RPA1. Thus, mutation of S15 would impair HR indirectly through an inhibitory effect on adjacent S37 phosphorylation.

The notion that p53 may suppress DSB repair has come from a series of studies looking at the effect of p53 on site-directed DSB in chromosomally integrated plasmid substrates [7], [12], [59]. We found that the magnitude of the suppressive p53 effect is correlated with the length of sequence homology present (Figure 2, S2). We postulate that the pDT219/pΔ2 system (Figure 2) is representative of sister chromatid repair because of the extent of available sequence homology is in the kilobase range. While we acknowledge that a comparison between different recombination systems has caveats, a dependence of p53's suppressive effect on homology length is in excellent agreement with a prior study by Wiesmüller et al. [12]. These authors, who used a panel of chromosomal EGFP-based substrates, demonstrated that the downregulation of gene conversion events by p53 was particularly pronounced when the length of shared homology was reduced to 168–233 bp. It is possible that p53 creates a threshold between short and long homologies, which may aid in preventing error-prone repair and detrimental rearrangements by misalignment of repetitive DNA. Such a model would be consistent with the observation that cellular p53 status has no direct effect on gene targeting and sister chromatid exchanges, which typically are mediated by long homologies in the order of kilobases [60]. This model also predicts that p53 will not negatively affect the repair of chromatid DSB caused by ionizing radiation or other agents, which is in line with cell survival data [61]. Our data also suggest that HR proficiency measured with an I-SceI based plasmid system such as pDR-GFP (Figure S2) is not always a good surrogate marker for HR-dependent repair of exogenous DNA damage. Further, recent data suggest that HR repair of chromosomal I-SceI-induced DSB is cell cycle dependent and subject to transcriptional regulation by p53 (Rieckmann et al., unpublished 2011). Thus, it is possible that HR activities in response to replication stress or frank DSB are differentially regulated by wild-type and mutant p53 variants. We can also not rule out that the regulatory effects may vary between cell lines.

Lastly, p53 did not compromise the RAD51 foci response and cell survival following exposure to the crosslinking agent MMC (Figure 6). To the contrary, there was even a slight increase in cell survival upon expression of transactivation-deficient p53 mutants (Figure 6E, S6). The underlying mechanism remains to be determined but may relate to a possible stabilization of multi-protein complexes at the replication fork by p53 (LMM, HW, unpublished data). The observed increase in MMC resistance is consistent with other reports showing that, in the absence of apoptosis, the presence of p53 is associated with cisplatin resistance [62], [63], [64]. In contrast, in cell systems or assays susceptible to apoptosis, resistance to DNA damaging agents is typically caused by loss of wild-type p53 [65], [66]. Overall, the role of p53 in determining cell survival in response to DNA damages is clearly complex and a reflection of p53's multiple functions in apoptosis, cell-cycle control, and DNA recombination. The study of these questions in defined cell systems is a promising avenue of investigation with potential clinical relevance for the treatment of malignant tumors most of which have lost p53 function. In addition, the biological significance of p53's function in HR regulation, especially with regard to its role in tumor suppression, remains to be established.

## Materials and Methods

### Cell lines

NCI-H1299 lung cancer cells (p53-null) were obtained from the ATCC and their use has been published previously [10]. A549 lung cancer cells were also obtained from the ATCC. Mouse embryonic fibroblasts (BALB/c 3T3 10.1 clone, p53-null) were a gift from Dr. Arnold Levine and their use has been published [14], [61]. H1299/FRT clones carrying different p53 mutants were generated according to the manufacturer's instructions (Flp-In, Invitrogen). Briefly, H1299 cells were electro-transfected with pFRT/LacZ followed by Zeocin (100 µg/ml, Invitrogen) selection to establish chromosomal integrants. Single-copy integrants with low transcriptional activity based on the co-integrated β-galactosidase reporter were selected for transfection with various pcDNA5/FRT-p53 constructs, concurrently with pOG44 for targeted integration into the FRT acceptor site. Following selection with 400 µg/ml Hygromycin (Invitrogen), colonies were expanded and whole cell lysates obtained to assess protein expression. For some experiments, H1299 cells carrying a randomly integrated p53QS construct (pRc/CMV- L22Q/W23S, kindly provided by Anindya Dutta) were used. MEFs were transfected with an expression vector for p53-A135V and selected with 500 µg/ml G418 (Fisher Scientific), as described previously [13]. All cell lines tested mycoplasma-free.

### RNA interference

ATR was targeted by a previously used and validated siRNA oligonucleotide, 5′- CCUCCGUGAUGUUGCUUGAtt -3′ (Applied Biosystems) [67]. Exponentially growing H1299 cells were plated 16 hours prior to transfection. ATR siRNA or a scrambled control was diluted in 100 µL Opti-MEM (Roche) to yield a final concentration of 100 nM, and mixed with 10 µL of X-tremeGENE transfection reagent (Roche) in 100 µL in Opti-MEM. Complex formation was allowed to proceed for 20 minutes at room temperature prior to drop wise addition to cells. siRNA complexes were incubated with cells for 4 hours, at which time cells were changed to normal growth media. The transfection was carried out twice, 24 hours apart, to achieve optimal knockdown. Whole cell lysates were obtained at 24 and 48 hours. Lysates were denatured and reduced, and then run on 3–8% Tris-Acetate gel (Invitrogen) for 2.5 hours at 150 V. The samples were transferred onto a PVDF membrane with a semi-dry apparatus (BioRad) for 1 hour at 12 V. The membrane was blocked in milk and then blotted with rabbit anti-ATR primary antibody (Bethyl Laboratories) at 1∶500 dilution at 4°C overnight followed by incubation with donkey anti-rabbit IgG HRP labeled secondary antibody (Amersham). Detection was performed with ECL reagents (Invitrogen) and the signal was developed on radiography films (GE Healthcare).

### Treatments

All treatments were carried out on exponentially growing cell populations in the absence of selection antibiotic. Thymidine and caffeine solutions (5 mM) were freshly prepared from 99% powder stock (Sigma-Aldrich) for each experiment. MMC (Sigma-Aldrich) was dissolved in water at 0.5 mg/ml and also used fresh. KU55933 (Chemdia) was dissolved in DMSO at 10 mM concentration, stored at −20°C, and used at a 10 µM working concentration.

### Plasmids

The N-terminal p53 mutants shown in Figure 3A were generated by modifying wild-type p53 cDNA, which had been cloned into pcDNA5/FRT [10], using site-directed mutagenesis (QuikChange II, Stratagene).

pDT219 carries a copy of the bacterial gpt gene inactivated by insertion of an I-SceI recognition site into the unique KpnI site of pSV2-gpt, as reported previously [54]. pΔ2, also reported previously [10], is characterized by 202 bp and 2333 bp of uninterrupted sequence homology shared with pDT217. To create pKEB1, the 198-bp fragment between the BglII and the EcoRV sites of pSV2-gpt was amplified using primers 5′ TAG TGC GCC AGA TCT CTA TA**C** TC**A** CGC GCA ACC TAT 3′ (forward, BglII underlined, base changes in bold) and 5′ GCG GGA TAT CAA CAA **TC**T AGT CAT CAA CCA GCG GAC 3′ (reverse, EcoRV underlined, base changes in bold). The gpt gene was inactivated by the indicated base changes 188 bp upstream and 250 bp downstream of the KpnI site and by creating an in-frame stop codon just upstream of the EcoRV site. In contrast to pΔ2, pKEB1 only contains 188 bp and 250 bp of homology flanking the break site.

The pCMV-I-SceI-3xNLS expression vector was kindly provided by Maria Jasin.

### HR reporter assay

MEFs were stably transfected with pDT219 using 1 µg/ml puromycin (Invitrogen), following our prior approach [13], and multiple clones were generated. Clones were co-transfected with pKEB1 or pΔ2 together with the I-SceI expression vector or a control and recombinants were scored in a colony formation assay based on XHATM selection as described [10], [13]. Transfection efficiencies were determined in parallel by transfection of pEGFP-N1 (Invitrogen) followed by FACS analysis.

### Immunofluorescence microscopy

Staining and visualization of RAD51 and γ-H2AX foci was performed using standard methods as described previously [54], [68]. RAD51 foci were visualized by incubating with anti-RAD51 antibody (PC130, Calbiochem) at 1∶200 dilution at 37°C for 3 hours. Gamma-H2AX was detected with an anti- γ-H2AX (phospho-S139) antibody (Ab18311, Abcam), incubating at 1∶200 dilution at 37°C for 1.5 hours. The number of foci per nucleus was routinely scored in a blinded fashion.

### Flow cytometry

Cell cycle distributions were determined using standard ethanol fixation and propidium iodide (Sigma-Aldrich) staining followed by flow cytometry, as described previously [13].

### Clonogenic cell survival assays

Colony formation assays were performed as previously published [61]. Following removal of MMC, cells were incubated for 2 weeks without selection antibiotic.

## Supporting Information

Figure S1 p53QS or wild-type p53 suppresses RAD51 foci formation in response to replication stress. (A) Impact of p53QS expression on thymidine-induced RAD51 foci in p53-null H1299 lung cancer cells. Cells were treated with either 1 mM hydroxyurea or 5 mm thymidine (both Sigma-Aldrich) for 24 hours and the number of RAD51 per nucleus was scored as shown. The difference in the number of induced foci (i.e., following subtraction of background foci numbers in untreated cells) becomes apparent when scoring cells with at least 10 foci per nucleus as positive. (B) Impact of endogenous wild-type (wt) p53 on thymidine (TdR)-induced RAD51 foci in A549 lung cancer cells compared to cells in which wt function was disrupted by stable transfection of a dominant-negative p53 mutant (mut) (expressed from the pC53-R273L plasmid vector). Y-axis represents percentage of cells with at least 10 induced RAD51 foci per nucleus, analogously to Figure 1.
(PDF)

Figure S2 Transactivation impaired p53 downregulates I-SceI induced HR in the pDR-GFP recombination substrate. (A) Plasmid substrate pDR-GFP carries two inactive copies of the enhanced green fluorescent protein (EGFP) (provided by Maria Jasin). Following I-SceI DSB induction, gene conversion is mediated by approximately 400 bp of uninterrupted shared homology flanking the break site. (B) I-SceI-induced HR frequencies obtained with chromosomally integrated pDR-GFP are plotted against p53 status in two isogenic cell pairs: H1299 cells (p53-null vector alone versus p53QS-transfected) and 10.1 mouse embryo fibroblasts (MEFs) (p53-null vector alone versus p53-A135V-transfected). Transient transfection of the pCMV-I-SceI-3xNLS expression vector or a control was followed by standard flow cytometry-based monitoring of recombinants. Induced HR events were corrected for spontaneous events and transient transfection efficiencies. Bars represent the geometric mean with SEM of at least 3 independent experiments. The relative suppression of HR in the presence of p53 compared to p53-null cells is indicated for each of the cell pairs. The relative suppression of HR was compared by the t-test (two-tailed).
(PDF)

Figure S3 Replication stress induces p53 serine 15 phosphorylation. (A) Whole cell lysates from H1299 cells incubated with 1 mM hydroxyurea (HU) or 5 mM thymidine (TdR) for 24 hours were obtained and subjected to incubation with a mouse polyclonal antibody against S15 phosphorylated p53 (16G8, #9286, Cell Signaling Technology) at 1∶1,000 dilution using standard immunoblotting methods. (B) S15 phosphorylation of p53 can be visualized as fine subnuclear foci at 100× magnification (anti-S15 phospho-p53, PC386, Calbiochem, at 1∶200 dilution). In this experiment, H1299 cells stably expressing low levels of p53QS were exposed to 24 hours of 0.1 mM HU. (C) Representative 40× images illustrate time course of S15 phosphorylation upon incubation of H1299 expressing p53QS with 1 mM HU.
(PDF)

Figure S4 p53 mutants were stably expressed in p53-null H1299 cells. Whole cell lysates from exponentially growing H1299 clones were obtained and subjected to incubation with a specific antibody against p53 (#9282, Cell Signaling Technology) using standard immunoblotting methods. The effect of the respective p53 status on HR activity is illustrated by arrows. Note that the p53 expression level in the clone expressing the p53-S15A mutant is somewhat lower even though it is expressed from the same chromosomal FRT acceptor site as the p53QS and p53QM mutants. The reason for this finding may be related to reduced protein stability but this was not pursued further. Generally, we have not found that the level of p53 expression affect the protein's ability to suppress HR (which is more of a function of local protein accumulation at DNA rather than overall expression in whole cell). For example, another H1299 clone which has the p53QS construct randomly integrated (marked by *) exhibits fully suppressed HR levels (shown in Figure 1B,C) even though the level of p53 protein expression is very low.
(PDF)

Figure S5 p53 downregulates RPA foci formation following replication stress. (A) Representative images illustrating the impact of p53 status on RPA foci in H1299 cells induced by 6 hours of thymidine (TdR) exposure (5 mM). RPA foci were visualized by first permeabilizing cells on ice with a buffer containing 0.5% Triton-X, 20 mM HEPES, 50 mM NaCl, 3 mM KCl, and 300 mM sucrose for 5 minutes, followed by fixing with 3% paraformaldehyde at room temperature for 30 minutes. Cells were stained with primary antibody against RPA (anti-RPA/p34, Thermo Scientific, MS-691-P0) at 1∶200 dilution for 3 hours at 37°C. (B) Quantification of foci counts. Y-axis represents percentage of treated cells with at least 10 induced foci, after subtracting the percentage of untreated cells with background RPA foci levels.
(PDF)

Figure S6 Transactivation impaired p53 promotes cellular resistance to mitomycin C (MMC). p53 null mouse embryonic fibroblasts with or without bi-allelic Rad54 knock-out (kindly provided by Fred Alt) were stable transfected with a plasmid vector encoding transactivation-deficient p53-A135V or an empty control. Survival was measured by standard colony formation and data points are based on 3–5 independent repeat experiments. p53-A135V promotes MMC resistance in a HR proficient background (left panel), similar to the p53 N-terminal mutants as shown in Figure 6E. Of note, loss of Rad54 function (right panel) appears to reduce cell survival only in the presence of p53 while in the absence of p53 loss of Rad54 has no effect on MMC sensitivity. It is possible that HR stimulation by p53 may overcome the impairment of HR caused by loss of Rad54 but this was not pursued further.
(PDF)
