# Supplementary figures and images for: ATR-p53 Restricts Homologous Recombination in Response to Replicative Stress but Does Not Limit DNA Interstrand Crosslink Repair in Lung Cancer Cells
Source: PLoS One. 2011 Aug 12;6(8):e23053. doi: 10.1371/journal.pone.0023053 (PMC3155521; doi:10.1371/journal.pone.0023053)

A

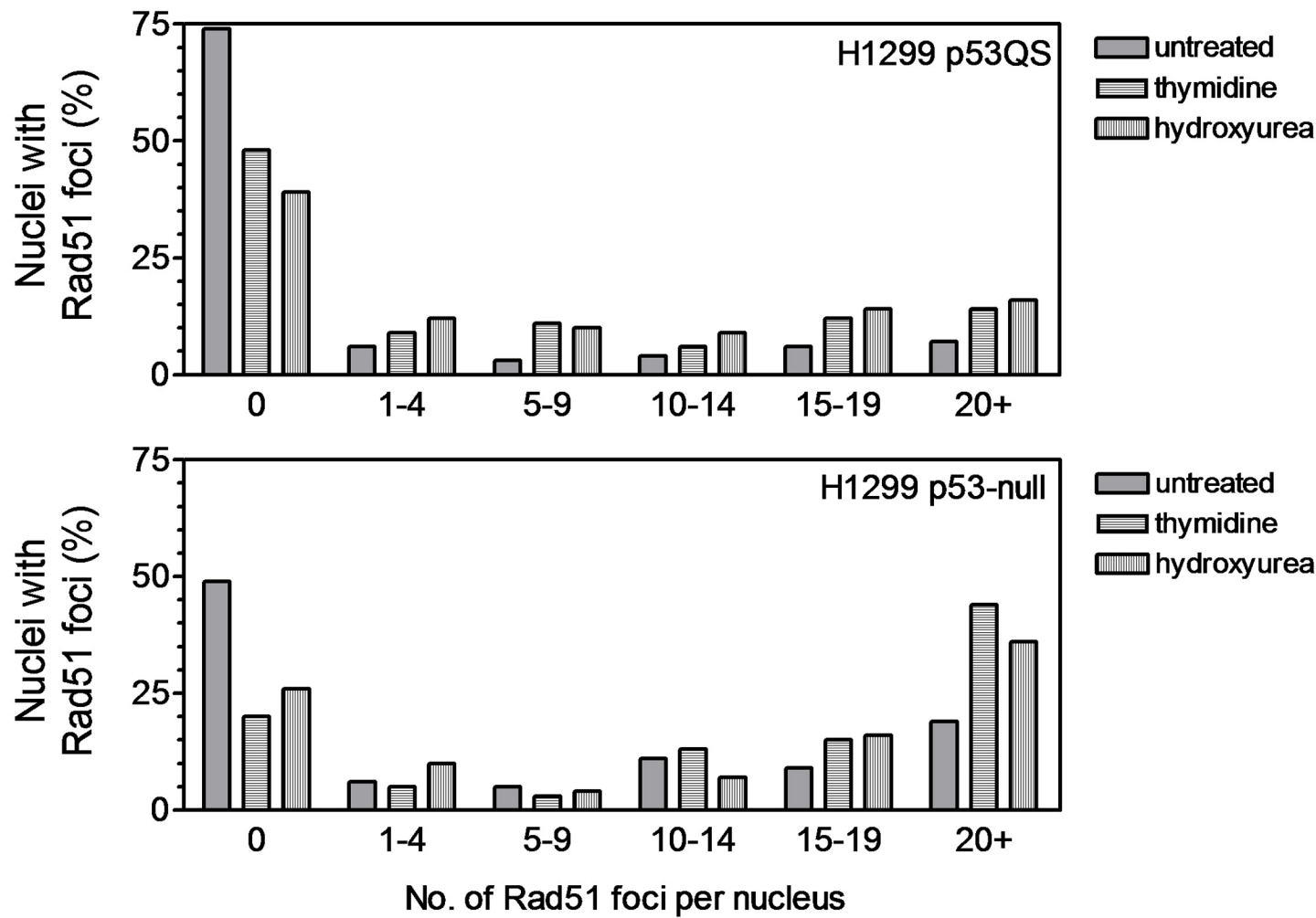

B

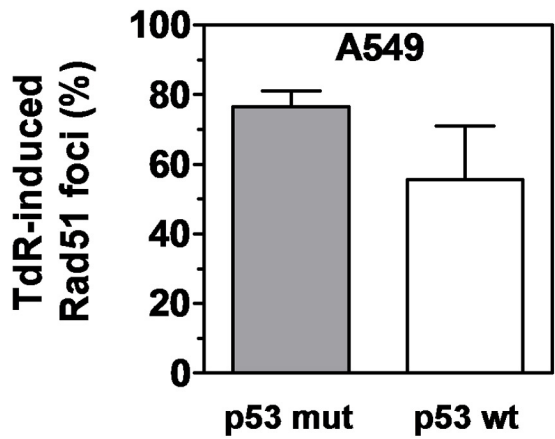

Supplement: Figure S1 — p53QS or wild-type p53 suppresses RAD51 foci formation in response to replication stress. (A) Impact of p53QS expression on thymidine-induced RAD51 foci in p53-null H1299 lung cancer cells. Cells were treated with either 1 mM hydroxyurea or 5 mm thymidine (both Sigma-Aldrich) for 24 hours and the number of RAD51 per nucleus was scored as shown. The difference in the number of induced foci (i.e., following subtraction of background foci numbers in untreated cells) becomes apparent when scoring cells with at least 10 foci per nucleus as positive. (B) Impact of endogenous wild-type (wt) p53 on thymidine (TdR)-induced RAD51 foci in A549 lung cancer cells compared to cells in which wt function was disrupted by stable transfection of a dominant-negative p53 mutant (mut) (expressed from the pC53-R273L plasmid vector). Y-axis represents percentage of cells with at least 10 induced RAD51 foci per nucleus, analogously to Figure 1. (PDF) [file pone.0023053.s001.pdf]

**A**

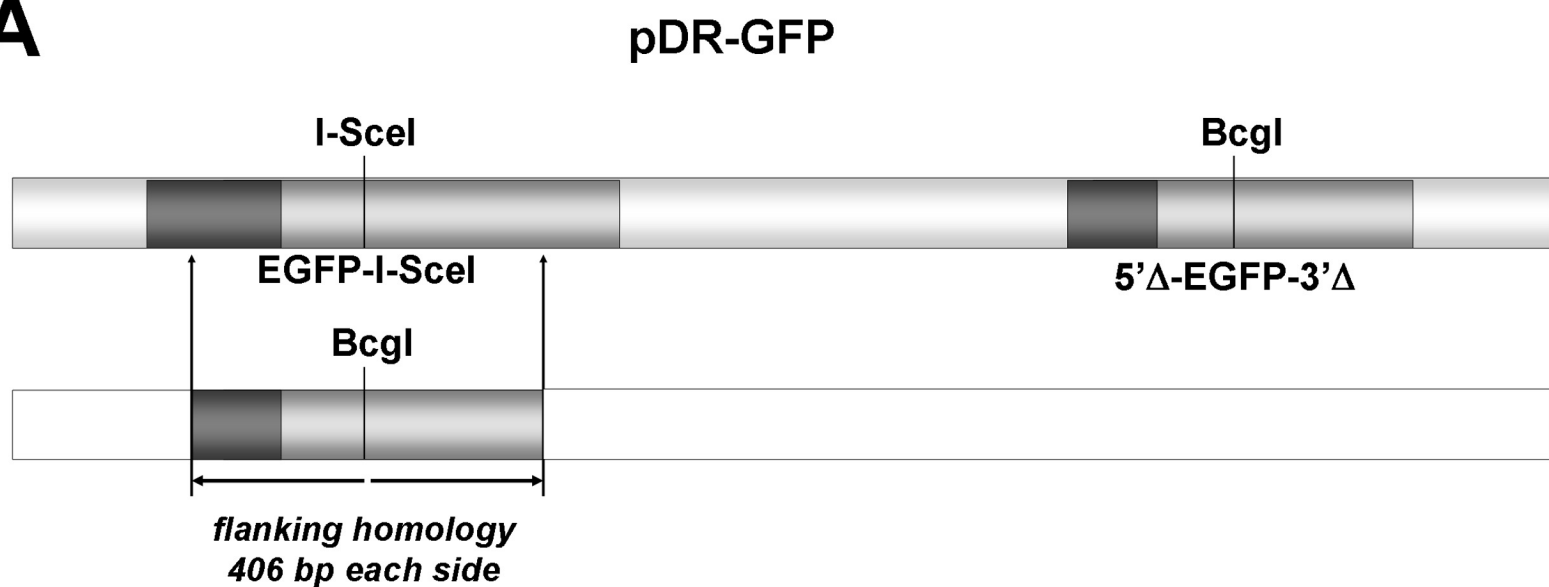

**B**

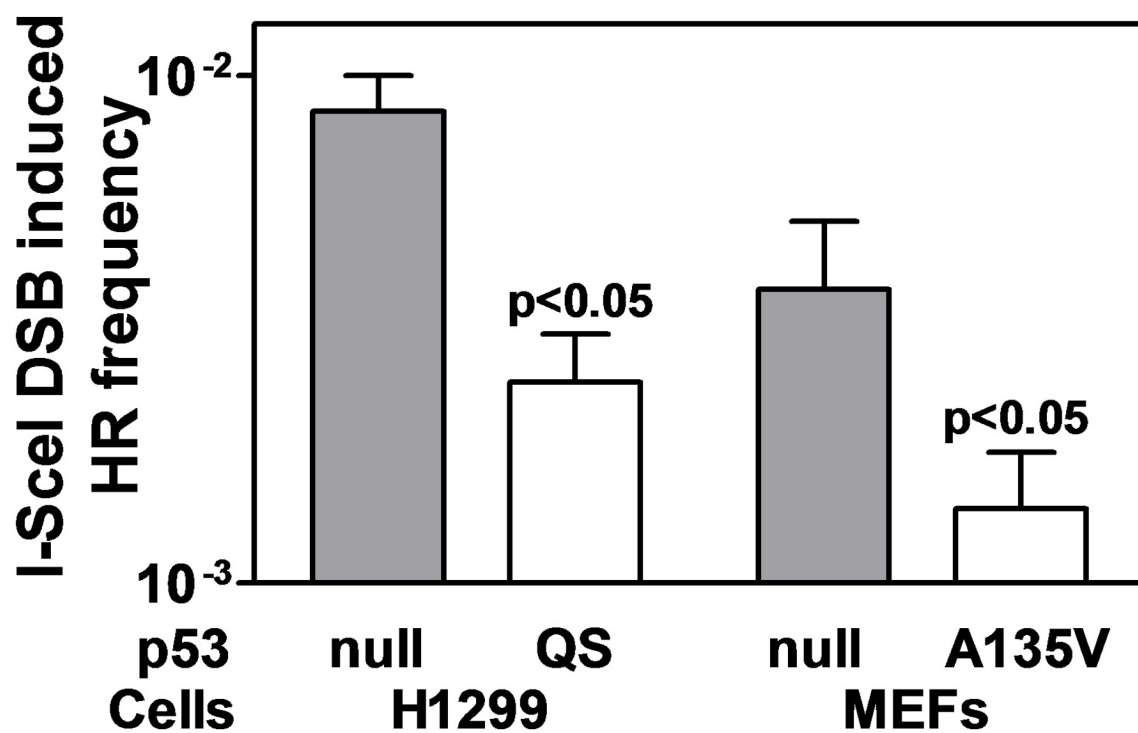

Supplement: Figure S2 — Transactivation impaired p53 downregulates I-SceI induced HR in the pDR-GFP recombination substrate. (A) Plasmid substrate pDR-GFP carries two inactive copies of the enhanced green fluorescent protein (EGFP) (provided by Maria Jasin). Following I-SceI DSB induction, gene conversion is mediated by approximately 400 bp of uninterrupted shared homology flanking the break site. (B) I-SceI-induced HR frequencies obtained with chromosomally integrated pDR-GFP are plotted against p53 status in two isogenic cell pairs: H1299 cells (p53-null vector alone versus p53QS-transfected) and 10.1 mouse embryo fibroblasts (MEFs) (p53-null vector alone versus p53-A135V-transfected). Transient transfection of the pCMV-I-SceI-3xNLS expression vector or a control was followed by standard flow cytometry-based monitoring of recombinants. Induced HR events were corrected for spontaneous events and transient transfection efficiencies. Bars represent the geometric mean with SEM of at least 3 independent experiments. The relative suppression of HR in the presence of p53 compared to p53-null cells is indicated for each of the cell pairs. The relative suppression of HR was compared by the t-test (two-tailed). (PDF) [file pone.0023053.s002.pdf]

# Suppl. Fig. S3

**A**

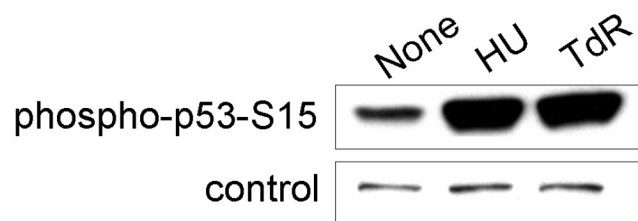

**B**

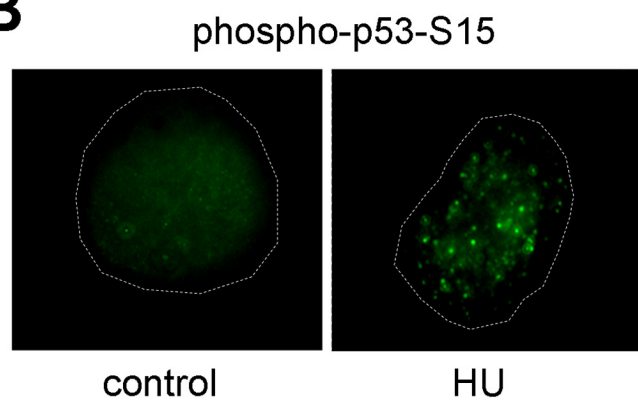

**C**

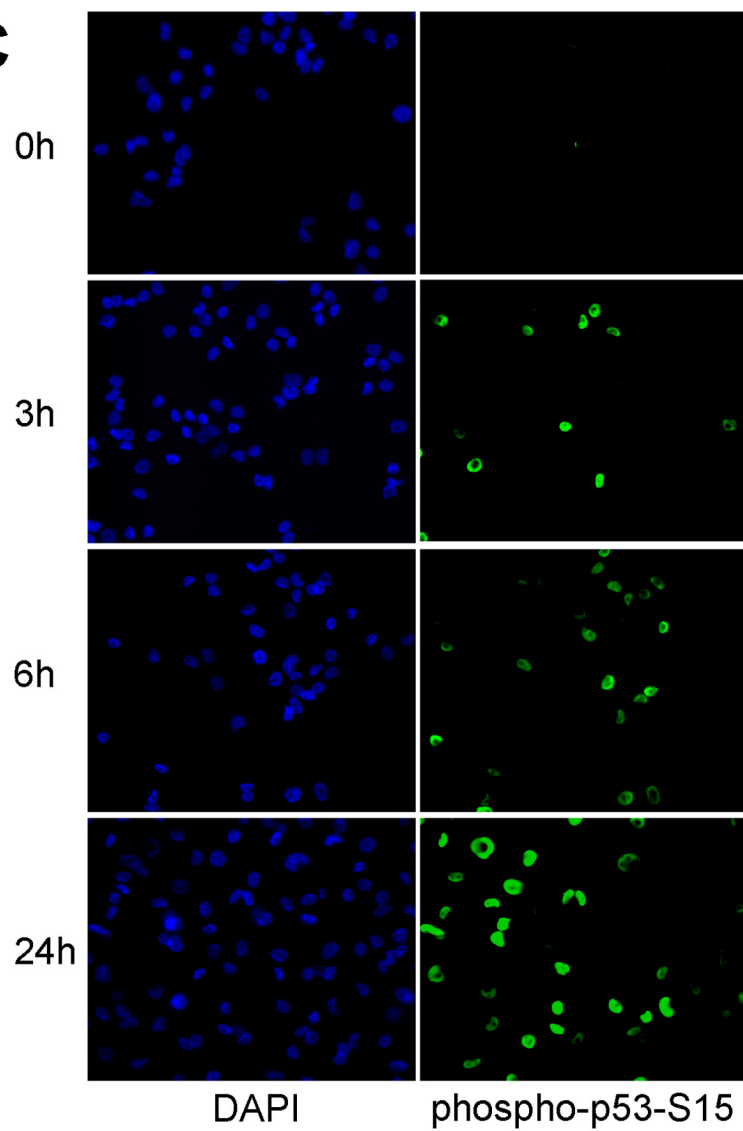

Supplement: Figure S3 — Replication stress induces p53 serine 15 phosphorylation. (A) Whole cell lysates from H1299 cells incubated with 1 mM hydroxyurea (HU) or 5 mM thymidine (TdR) for 24 hours were obtained and subjected to incubation with a mouse polyclonal antibody against S15 phosphorylated p53 (16G8, #9286, Cell Signaling Technology) at 1∶1,000 dilution using standard immunoblotting methods. (B) S15 phosphorylation of p53 can be visualized as fine subnuclear foci at 100× magnification (anti-S15 phospho-p53, PC386, Calbiochem, at 1∶200 dilution). In this experiment, H1299 cells stably expressing low levels of p53QS were exposed to 24 hours of 0.1 mM HU. (C) Representative 40× images illustrate time course of S15 phosphorylation upon incubation of H1299 expressing p53QS with 1 mM HU. (PDF) [file pone.0023053.s003.pdf]

Suppl. Fig. S4

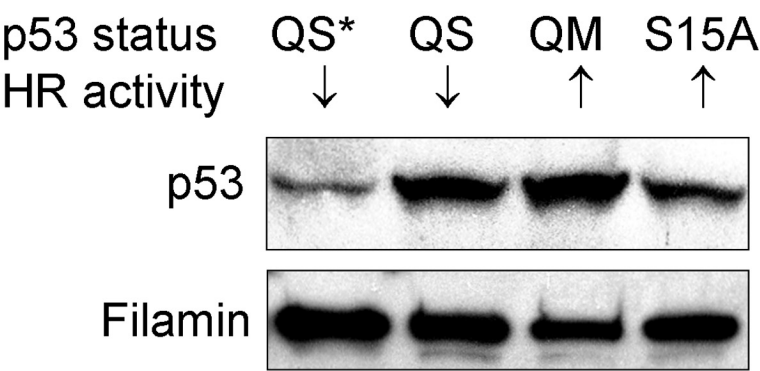

Supplement: Figure S4 — p53 mutants were stably expressed in p53-null H1299 cells. Whole cell lysates from exponentially growing H1299 clones were obtained and subjected to incubation with a specific antibody against p53 (#9282, Cell Signaling Technology) using standard immunoblotting methods. The effect of the respective p53 status on HR activity is illustrated by arrows. Note that the p53 expression level in the clone expressing the p53-S15A mutant is somewhat lower even though it is expressed from the same chromosomal FRT acceptor site as the p53QS and p53QM mutants. The reason for this finding may be related to reduced protein stability but this was not pursued further. Generally, we have not found that the level of p53 expression affect the protein's ability to suppress HR (which is more of a function of local protein accumulation at DNA rather than overall expression in whole cell). For example, another H1299 clone which has the p53QS construct randomly integrated (marked by *) exhibits fully suppressed HR levels (shown in Figure 1B,C) even though the level of p53 protein expression is very low. (PDF) [file pone.0023053.s004.pdf]

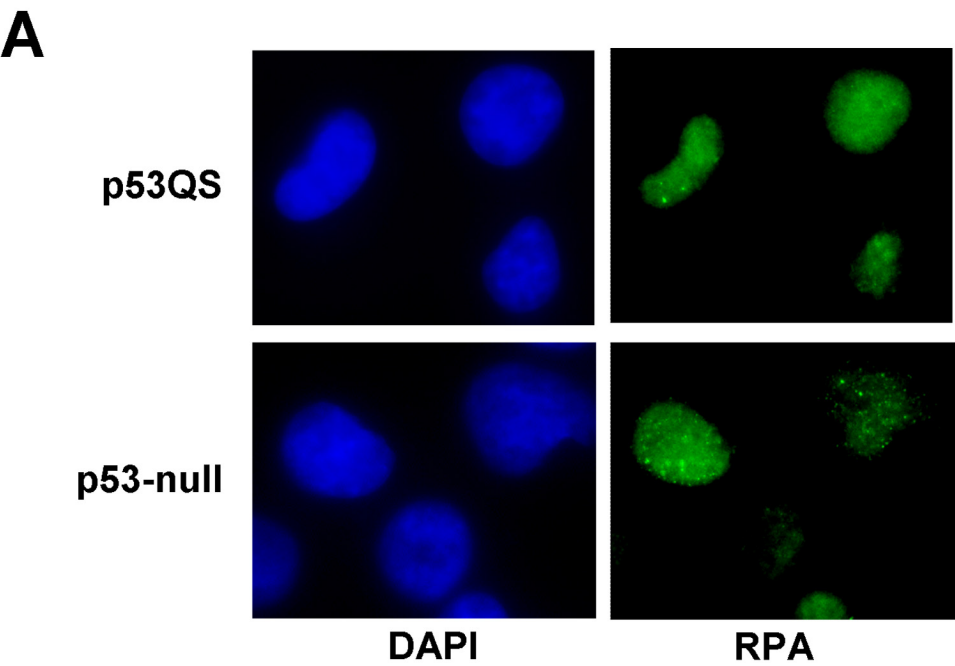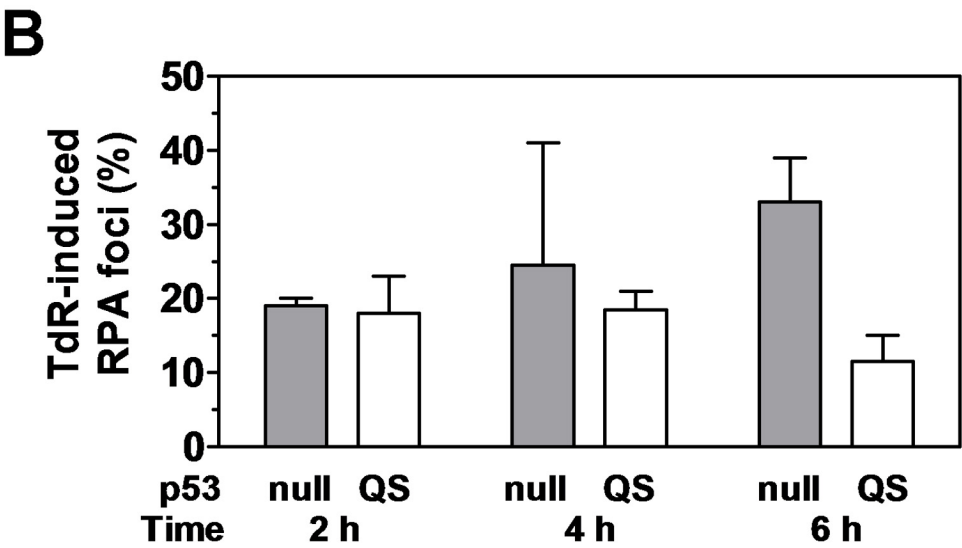

Supplement: Figure S5 — p53 downregulates RPA foci formation following replication stress. (A) Representative images illustrating the impact of p53 status on RPA foci in H1299 cells induced by 6 hours of thymidine (TdR) exposure (5 mM). RPA foci were visualized by first permeabilizing cells on ice with a buffer containing 0.5% Triton-X, 20 mM HEPES, 50 mM NaCl, 3 mM KCl, and 300 mM sucrose for 5 minutes, followed by fixing with 3% paraformaldehyde at room temperature for 30 minutes. Cells were stained with primary antibody against RPA (anti-RPA/p34, Thermo Scientific, MS-691-P0) at 1∶200 dilution for 3 hours at 37°C. (B) Quantification of foci counts. Y-axis represents percentage of treated cells with at least 10 induced foci, after subtracting the percentage of untreated cells with background RPA foci levels. (PDF) [file pone.0023053.s005.pdf]

Suppl. Fig. S6

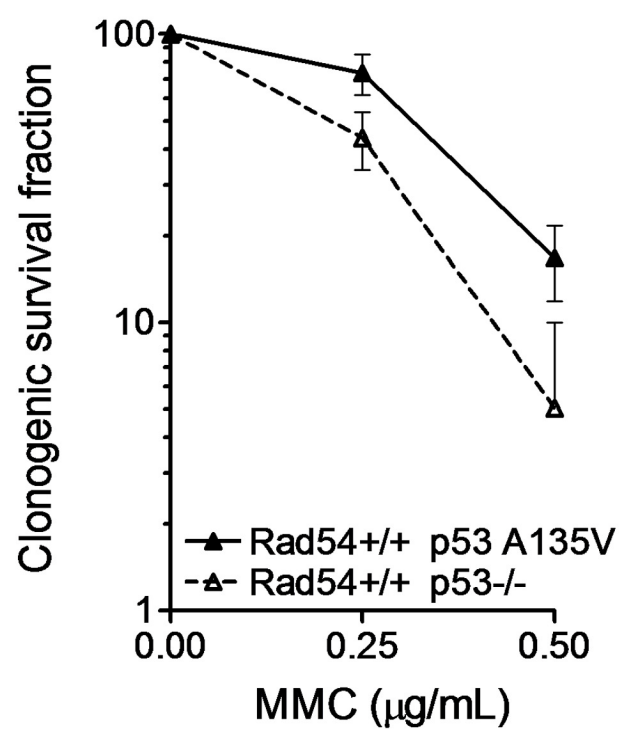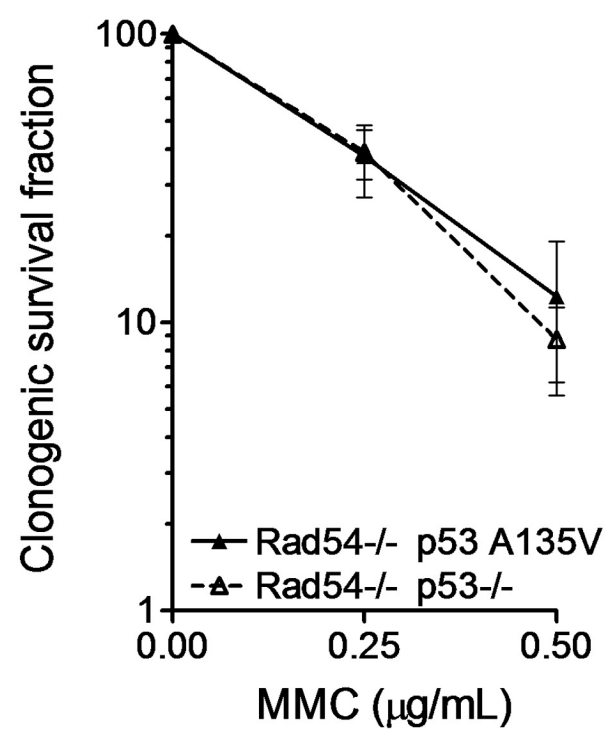

Supplement: Figure S6 — Transactivation impaired p53 promotes cellular resistance to mitomycin C (MMC). p53 null mouse embryonic fibroblasts with or without bi-allelic Rad54 knock-out (kindly provided by Fred Alt) were stable transfected with a plasmid vector encoding transactivation-deficient p53-A135V or an empty control. Survival was measured by standard colony formation and data points are based on 3–5 independent repeat experiments. p53-A135V promotes MMC resistance in a HR proficient background (left panel), similar to the p53 N-terminal mutants as shown in Figure 6E. Of note, loss of Rad54 function (right panel) appears to reduce cell survival only in the presence of p53 while in the absence of p53 loss of Rad54 has no effect on MMC sensitivity. It is possible that HR stimulation by p53 may overcome the impairment of HR caused by loss of Rad54 but this was not pursued further. (PDF) [file pone.0023053.s006.pdf]
